# Causes of mortality in laying hens in different housing systems in 2001 to 2004

**DOI:** 10.1186/1751-0147-51-3

**Published:** 2009-01-15

**Authors:** Oddvar Fossum, Désirée S Jansson, Pernille Engelsen Etterlin, Ivar Vågsholm

**Affiliations:** 1 Department of Animal Health and Antimicrobial Strategies, National Veterinary Institute (SVA), SE-751 89 Uppsala, Sweden; 2 Department of Pathology and Wildlife Diseases, National Veterinary Institute (SVA), SE-751 89 Uppsala, Sweden; 3 Office of Science and Quality, National Veterinary Institute (SVA, SE-751 89 Uppsala, Sweden

## Abstract

**Background:**

The husbandry systems for laying hens were changed in Sweden during the years 2001 – 2004, and an increase in the number of submissions for necropsy from laying hen farms was noted. Hence, this study was initiated to compare causes of mortality in different housing systems for commercial laying hens during this change.

**Methods:**

Based on results from routine necropsies of 914 laying hens performed at the National Veterinary Institute (SVA) in Uppsala, Sweden between 2001 and 2004, a retrospective study on the occurrence of diseases and cannibalism, i.e., pecking leading to mortality, in different housing systems was carried out. Using the number of disease outbreaks in caged flocks as the baseline, the expected number of flocks with a certain category of disease in the other housing systems was estimated having regard to the total number of birds in the population. Whether the actual number of flocks significantly exceeded the expected number was determined using a Poisson distribution for the variance of the baseline number, a continuity correction and the exact value for the Poisson distribution function in Excel 2000.

**Results:**

Common causes of mortality in necropsied laying hens included colibacillosis, erysipelas, coccidiosis, red mite infestation, lymphoid leukosis and cannibalism. Less common diagnoses were Newcastle Disease, pasteurellosis and botulism. Considering the size of the populations in the different housing systems, a larger proportion of laying hens than expected was submitted for necropsy from litter-based systems and free range production compared to hens in cages (*P *< 0.001). The study showed a significantly higher occurrence of bacterial and parasitic diseases and cannibalism in laying hens kept in litter-based housing systems and free-range systems than in hens kept in cages (*P *< 0.001). The occurrence of viral diseases was significantly higher in indoor litter-based housing systems than in cages (*P *< 0.001).

**Conclusion:**

The results of the present study indicated that during 2001–2004 laying hens housed in litter-based housing systems, with or without access to outdoor areas, were at higher risk of infectious diseases and cannibalistic behaviour compared to laying hens in cages. Future research should focus on finding suitable prophylactic measures, including efficient biosecurity routines, to reduce the risk of infectious diseases and cannibalism in litter-based housing systems for laying hens.

## Background

The Swedish Animal Welfare Act from 1988 mandated a switch from conventional battery cages for laying hens to alternative housing systems i.e. litter-based housing systems, including free range, and enriched cages. A fundamental requirement according to the new Swedish legislation was that laying hens should be able to behave naturally, e.g. have access to nests, perches and dust baths.

The change of housing systems started slowly, but during the years 2001–2004 practically all the remaining conventional battery cages, equivalent to about 80 % of the population of layers in 2001, were exchanged for alternative housing systems. During the same period, the number of routine necropsies of laying hens submitted to The National Veterinary Institute (SVA) in Uppsala, Sweden increased noticeably compared to the years before. Results from necropsies from this period have for that reason been compiled in an effort to analyse the effects of the change in housing systems on bird health. The results will probably have relevance outside Sweden, as a decision has been taken in the European Union to ban conventional battery cages by 2012 [1].

## Methods

The study was based on results from necropsies of commercial laying hens submitted to SVA for routine examinations from all parts of Sweden in connection with disease associated with increased mortality in the flocks. Other diseases, parasite infections or lesions not leading to a manifest increase of mortality are not included in this study. During the study period (January 1, 2001 to December 31, 2004) four different hybrids were used; Lohmann Selected Leghorn, Lohmann Brown, Hy-Line White and Hy-Line Brown. At the time of the study, laying hens in Sweden were vaccinated against Marek's disease, avian encephalomyelitis and infectious bronchitis during the rearing period and against infectious bronchitis regularly during the laying period. Most laying hens intended for litter-based housing systems were also immunised against coccidiosis early during the rearing period.

In Sweden, there were three veterinary diagnostic laboratories, including SVA, which performed necropsies on farm animals during the study period. Numbers of poultry necropsies performed by the other laboratories (Kallay TB and Linder A, AnalyCen Nordic AB, personal communication) were compared to those of SVA. The share of SVA was 78% of all chicken necropsies during the period 2001 to 2004. During the study period, SVA's share of laying hen necropsies remained stable.

The criteria for inclusion in this study were the following: 1) The laying hens must come from a flock with increased mortality. 2) The number of submitted hens should be three or more. 3) There should be information about the housing system, age of the birds, flock size, and disease history in the flock. All birds were necropsied according to a routine protocol of SVA. Histological, bacteriological, virological, parasitological and chemical examinations were performed having regard to disease history and gross pathological findings. These examinations were performed according to routine methods used at SVA. In some cases additional diagnostic tests were required such as PCR and virus isolation for Newcastle Disease virus, and serology to detect antibodies against avian leukosis virus. The protocols for necropsy, microbiological and chemical examinations (in Swedish) are available from the corresponding author upon request.

The housing systems used in Sweden during the study period were: 1) single-tiered floor systems with manure bins, with or without regular removal of manure, 2) multi-tiered floor systems (aviaries) with litter belts for regular removal of manure, 3) conventional battery cages, and 4) furnished cages (cages with perches, nests and dust bathing areas). A review of housing systems in use is given by R. Tauson [2]

In the single-tiered floor systems the maximum bird density allowed was 7.5 or 9 birds per m^2 ^available area (depending on the body weight) while the maximum density in aviaries was 7 birds per m^2 ^available area or 20 birds per m^2 ^floor area. The required area in single nests was 0.0125 m^2 ^per bird and in colony nests 0.010 m^2 ^per bird. For all housing systems at least 15 cm perch per hen was required. The conventional cages should according to the regulations in force during the time of the study offer an available area of at least 600 cm^2 ^per bird while furnished cages in addition should have a nest and a dust bathing area of 200 cm^2^each [3]. Ten birds per cage was the largest group size in furnished cages at the time of the study. Hens in free-range systems, including organic laying hens, were housed in either single-tiered floor systems or multi-tiered aviary systems, and the birds had access to outside pens and/or pasture. In organic production the maximal number of hens allowed during the study period was 7 per m^2 ^available floor area [4].

Submitted birds were divided into three categories: 1) birds housed in conventional and furnished cages 2) birds housed indoors in litter-based housing systems (single-tiered floor systems and multi-tiered aviary systems), and 3) birds in free range systems with access to outdoor pens and/or pasture, including organic laying hens. Since the numbers of submissions from conventional cages and furnished cages were small we considered birds from these two systems as one category. Birds from indoor single-tiered floor systems and multi-tiered aviary systems were also regarded as one category since the available annual reports showing the hen populations in Sweden made no distinction between these systems. These two systems are henceforward called litter-based systems. The sizes of flocks included in the study varied from 400 birds to 37,000 birds, and the age of the laying hens ranged from 18 to 78 weeks.

During the study period there was a change in housing systems for Swedish laying hens with a decrease of hens in conventional cage systems and an increase of hens in enriched cages and in litter-based housing systems including organic production. The total number of laying hens per year and the proportions of hens in different housing systems [5] are presented in Table 1.

**Table 1:** Percentage of commercial laying hens in different housing systems in Sweden in 2001–2004^1^. The total number of hens varied between 5,042 to 5,904 million birds per year.

**Year/Housing system**	**2001**	**2002**	**2003**	**2004**	**2001–2004**
**Conventional battery cages**	66.5	52.3	26.0	13.5	38.6
**Furnished cages**	7.5	12.6	24.2	24.8	17.6
**Litter-based systems^2^**	22.6	31.2	43.8	55.0	38.7
**Free range^3^**	3.4	3.9	6.0	6.7	5.1

**Total**	100	100	100	100	100

^1^The table is based on data collected in May to August each year [5].

^2^Includes single-tiered floor systems and multi-tiered aviary systems.

^3^Flocks housed in litter-based systems indoors, with access to outdoor pens and pasture, including organic flocks.

The length of the egg production period for laying hens in Sweden during the study period was approximately 12 months. For the analysis, we therefore assumed that approximately four production periods were completed for each category during the four-year period. The categories of populations that we compared were the total number of birds in each of the main housing systems (cages, litter based systems and free range) during the four-year period.

### Statistical analysis

In this study, the epidemiological units of interest were the flocks from which birds were submitted for necropsy. Thus, the effects of variable numbers of birds in some of the submissions and of repeated submissions from some flocks were reduced. The results of necropsies were divided into major diagnostic categories i.e. bacterial, viral, parasitic diseases, and cannibalism. As more than one diagnosis was observed in some of the flocks, these flocks were represented in two or more diagnostic categories. On the other hand, several submissions from one single flock showing the same disease were considered as a single "outbreak". Using flocks of laying hens kept in cages from which birds were submitted for necropsy as the baseline, the expected number of flocks with submissions with each of the major diagnoses was estimated for indoor litter based systems and for free-range systems. We assumed that the number of flocks would be proportionate to the total number of birds in the different systems during the four year period. Since the submission of laying hens for necropsy was a rare occasion given the total number of birds, we chose to compare the number of flocks from indoor litter based systems with cage systems, and free-range systems with cage systems. Whether these numbers significantly exceeded the expected numbers was determined looking at the upper 95% confidence level for the expected number assuming a Poisson distribution for the variance of the expected baseline number, a continuity correction, and the exact value for the Poisson distribution function in Excel 2000 [6].

## Results

### Causes of mortality

Altogether 914 laying hens from 172 flocks were included in the study (Table 2). In most submissions one specific disease was predominant i.e. was observed in more than 50% of the submitted laying hens. Less common were cases in which two (or more) diseases contributed substantially to the mortality in a flock (observed in 12% of the flocks) which was usually noticed when birds were submitted from the same flock several times with long intervals. The main disease categories of the included flocks are given in Table 3. Diseases or problems of other aetiologies than mentioned in the table were infrequent, and were usually not the main cause of increased mortality on flock level at the time of investigation. A summary of these findings is presented in Table 4.

**Table 2:** Numbers and percentage of commercial laying hen flocks submitted for necropsy to SVA and included in the study.

	**2001**		**2002**		**2003**		**2004**	
**Housing systems**	***N***	**%**	***N***	**%**	***N***	**%**	***N***	**%**

**Cages^1^**	5	41.7	6	9.1	4	9.5	5	9.6
**Litter-based systems^2^**	5	41.7	54	81.8	32	76.2	38	73.1
**Free range^3^**	2	16.6	6	9.1	6	14.3	9	17.3

**Total**	12	100	66	100	42	100	52	100

^1^Includes conventional battery cages and enriched cages.

^2^Includes single-tiered floor systems and multi-tiered aviary systems.

^3^Flocks housed in litter-based systems indoors, with access to outdoor pens and pasture, including organic flocks.

**Table 3:** Occurrence of diseases (divided into main categories) in laying hen flocks submitted to SVA in 2001–2004.

		**Bacterial diseases**		**Viral**	**diseases**	**Parasitic diseases**		**Cannibalism**	
**Housing system**	**Total no. of flocks^1^**	***N***	**%**	***N***	**%**	***N***	**%**	***N***	**%**
**Cages^2^**	20	13	65.0	6	30.0	2	10.0	1	5.0
**Litter-based systems^3^**	129	94	72.9	15	11.6	23	17.8	24	18.6
**Free range^4^**	23	17	73.9	1	4.4	5	21.7	6	26.1

^1^No. of examined flocks. Note that each flock may have several diagnoses.

^2^Includes conventional battery cages and enriched cages.

^3^Includes single-tiered floor systems and multi-tiered aviary systems.

^4^Flocks housed in litter-based systems indoors, with access to outdoor pens and pasture, including organic flocks.

**Table 4:** List of miscellaneous diagnoses in 173 necropsied laying hens, not included in main diagnostic categories and the statistical analysis.

		**Housing of the birds (no. of flocks)**		
		
**Diagnosis**	**No. of hens**	**Cages^1^**	**Litter-based systems^2^**	**Free range^3^**
Visceral gout	29	1	10	1
Unknown cause of death	23	6	17	0
Dehydration and cachexia	17	0	6	0
Trauma	16	2	12	2
Hepatic lipidosis and liver rupture	11	3	2	0
Circulatory disturbances^4^	16	0	3	0
Anaemia and haemorrhages, suspected intoxication	11	0	0	1
Osteoporosis	10	4	0	0
Egg bound	7	3	4	0
Hepatitis	5	0	4	1
Obstipation (straw)	5	0	1	2
Miscellaneous neoplasms^5^	4	1	3	0
Other causes of death^6^	19	2	17	0

^1^Includes conventional battery cages and enriched cages.

^2^Includes single-tiered floor systems and multi-tiered aviary systems.

^3^Flocks housed in litter-based systems indoors, with access to outdoor pens and pasture, including organic flocks.

^4^Botulism was later diagnosed from hens in the same flocks.

^5^Neoplasms other than lymphoid leukosis.

^6^Including egg yolk peritonitis (*n *= 4), gastritis (*n *= 2), ovarian cyst (*n *= 2), air sacculitis (*n *= 2), enteritis (*n *= 2), chronic salpingitis (*n *= 1), endocarditis (*n *= 1), intestinal volvulus (*n *= 1), myositis (*n *= 1) ruptured ovary and abdominal hemorrhage (*n *= 2). hepatitis (*n *= 1).

### Bacterial diseases

Bacterial diseases appeared to be the most common causes of mortality during the study period (Table 3). The predominating disease in all housing systems was infections caused by *Escherichia *(*E*.) *coli*, i.e. colibacillosis, which was found in 85 flocks. The pathological findings were usually acute or subacute fibrinous salpingitis, oophoritis and peritonitis, or more infrequently pericarditis, perihepatitis, pneumonia and air sacculitis. A relatively high proportion (52%) of the cases of colibacillosis occurred between start of lay and 30 weeks of age. In 50% of the infected flocks cloacal cannibalism or vent pecking was observed in one or more birds submitted for necropsy. Severe cloacal injuries were observed in 19% (out of 312) of the layers with colibacillosis.

Outbreaks of erysipelas, caused by *Erysipelothrix *(*E*.) *rhusiopathiae*, were observed in ten of 129 flocks from indoor litter-based housing systems and in six of 23 flocks from free-range systems but not in any caged flock. The post mortem examinations showed birds with acute, septicaemic disease with splenomegaly and hepatomegaly as the most prominent lesions. Valvular endocarditis, necrotic hepatitis, and necrotic splenitis were observed sporadically.

In four flocks kept in litter-based systems indoors, infections with *Pasteurella multocida *were diagnosed. No cases of pasteurellosis were observed in caged birds or free-range birds.

Botulism type C/D was diagnosed on one farm with laying hens kept in aviaries. The birds had shown signs of paralysis and high mortality. Birds from two houses on the farm were submitted for necropsy. Signs of circulatory disturbances/failure such as pale musculature and congested abdominal organs were observed in most of the laying hens and no other gross or microscopic lesions were noted.

### Parasitic diseases

Coccidiosis was observed in 18 of 129 flocks housed in litter-based systems indoors, in five of 23 free-range flocks and in two of 20 flocks from cage systems. Most of the outbreaks (76%) occurred in birds younger than 24 weeks.

In five of 129 flocks kept in indoor litter-based housing systems, increased mortality caused by infestation with the poultry red mite (*Dermanyssus *(*D*.) *gallinae*) was demonstrated. In addition, *D*. *gallinae *were found in the plumage of birds from another seven flocks from litter-based housing systems including free range. Mites were not detected in birds submitted from cages.

### Viral diseases

In total, 22 outbreaks of viral diseases were diagnosed within the frame of the study. Lymphoid leukosis was diagnosed in ten of 129 flocks housed in litter based housing systems, and in six of 20 flocks housed in cages. These outbreaks were observed in birds in the age interval 20–39 weeks during 2001 and 2002. No case of lymphoid leukosis was observed in free-range birds. Marek' s disease was diagnosed in two of 129 flocks in litter-based housing systems and in one of 23 free-range flocks during 2001–2002. Newcastle Disease was diagnosed 2004 in three flocks housed in litter-based housing systems indoors from two closely situated, but separate farms.

### Cannibalism

The most common traumatic injuries in the necropsied birds leading to increased mortality were wounds in the cloacal region indicative of vent pecking (cannibalism). Cannibalism was observed in all housing systems and was the main cause of mortality in five of 129 flocks housed in litter based systems indoors and in four of 23 free range flocks. Cannibalism was not observed as the main cause of death in any of the flocks housed in cages.

### Differences in occurrence of diseases between housing systems

The results from the statistical analyses are shown in tables 5 and 6.

**Table 5:** Risk analysis regarding main disease categories between laying hen flocks in cages^1 ^and litter-based systems^2^.

**Disease category**	**Observed no. of flocks from litter-based systems**	**Expected no. of flocks if same risk as in cages**	**Excess no. of flocks in litter-based systems compared to cages**
**Bacterial diseases**	94	9	85***
**Viral diseases**	15	4	11***
**Parasitic diseases**	23	1	22***
**Cannibalism**	24	1	23***

**Total**	129	14	115***

^1^Includes conventional battery cages and enriched cages.

^2^Includes single-tiered floor systems and multi-tiered aviary systems.

Level of significance is denoted by *** *P *< 0.001.

**Table 6:** Risk analysis regarding main disease categories between laying hen flocks in cages^1 ^and free range housing^2^.

**Disease category**	**Observed no. of flocks from free range housing**	**Expected no. of flocks if same risk as in cages**	**Excess no. of flocks in free range housing compared to cages**
**Bacterial diseases**	17	1	16***
**Viral diseases**	1	1	0
**Parasitic diseases**	5	0	5***
**Cannibalism**	6	0	6***

**Total**	23	2	21***

^1^Includes conventional battery cages and enriched cages.

^2^Flocks housed in litter-based systems indoors, with access to outdoor pens and pasture, including organic flocks.

Levels of significance are denoted by *** *P *< 0.001

## Discussion

The results of the present study showed that there was a significant difference between housing systems concerning the diagnostic categories and number of flocks from which laying hens were submitted for necropsy to SVA during 2001–2004. Compared to caged flocks, more submissions than expected arrived for necropsy from indoor litter-based housing systems and from farms with hens in free-range systems. Supporting evidence for assuming that the presented results reflected the true health situation of Swedish laying hens during the time of study would be: 1) That the readiness of the poultry farmers to submit laying hens for necropsy was independent of their choice of housing system, and 2) That the farms with different housing systems were evenly distributed in the country. Based on our long experience from routine diagnostics, we cannot exclude that there may be differences in the owners´ willingness to submit birds for necropsy from different housing systems. Available information indicates, however, that the primary cause of the increase of submissions to SVA during the study period were health problems connected to the change in housing systems as there were no other known major changes in the egg production industry during that time. Furthermore, the cost of necropsies remained stable and the market share of SVA did not change substantially. In the second case, regional differences were known to exist, i.e. in some areas of the country litter-based housing systems were more common than in other regions. However, SVA received laying hens for necropsy from all regions of the country, which to some extent reduced the risk for biases due to regional differences.

Bacterial diseases were the most common causes of mortality in laying hens submitted to SVA for necropsy. Similar results have been reported from surveys on health of commercial layers in other countries [7-9]. Bacterial infections seem to be more common in laying hens in litter-based housing systems including free-range birds, than in caged birds [8,10]. Furthermore, *Kreienbrock et al*. (2003) found that antibiotics were more commonly used in German laying hens kept in litter-based housing systems than in caged birds [10].

The most common bacterial infection diagnosed in this study was colibacillosis, which has been reported from many countries as a frequent cause of disease in commercial laying hens, as well as in hens in experimental trials [7,9,11-14]. The dominating lesions observed in our cases were in agreement with reports from other countries [12-15]. An interesting finding in this study was that a high proportion of the birds infected with *E*. *coli *showed wounds or purulent inflammation in the cloacal region, which indicates that vent pecking was an important predisposing factor. Mortality caused by cannibalism was also common in these flocks. Similar observations have been reported from commercial flocks, as well as from experimental trials [9,16-18].

Infections with *E. rhusiopathiae *and *P. multocida *were observed in hens in litter-based systems, with or without free-range, but not from hens in cages. Erysipelas has previously been reported sporadically in laying hens in different housing systems from several countries [8,19-22]. Our finding concur with reports from Germany indicating that there is a higher risk of erysipelas in laying hens housed in litter-based housing systems (including free-range) than in caged birds [10,19].

The first case of botulism in laying hens in Sweden was diagnosed in hens housed in an aviary system in 2003. Worldwide, reports of botulism in laying hens are very rare with only a few outbreaks reported in the literature [23,24], whereas the disease has more often been observed in broilers [25]. *Huin and Sakaguchi *showed that keeping broilers on litter increased the risk of botulism compared to housing in cages [26], and this may explain the difference between laying hens and broilers concerning the number of reports in recent years. The Swedish outbreak of botulism may indicate an increased risk associated with litter-based housing systems for laying hens.

This study showed an apparent difference between litter-based housing systems and cages concerning submissions due to coccidiosis. During the study period many commercial pullets were reared in cages because of a shortage of litter-based rearing systems. The fact that these cage-reared birds usually were not vaccinated against coccidiosis may explain many of the outbreaks during the study period. However, it cannot be excluded that suboptimal administration of the vaccine may explain some of the cases. Probably, the risk of coccidiosis can be further reduced by adjusting the way pullets are reared to the conditions they meet in the production units. It is also very important to ensure that the vaccination of pullets intended to produce in litter based systems offers sufficient immunity.

The poultry red mite, *D*. *gallinae*, caused increased mortality in several flocks in the litter-based systems. This parasite is widespread on poultry farms in Europe [27-32], and mortality as a consequence of mite infestation occurs sporadically [27,28,30,31]. However, the most common effects of mite infestation are the reduced welfare of the birds and the economic loss due to reduced egg production [29,31]. Free range systems have been reported to be more commonly affected by mites than other housing systems [30,32]. Kreienbrock et al. 2003, observed a more frequent use of acaricides in German laying hen farms with litter-based systems than in farms with cages [10] which concur with our results.

With the exception of lymphoid leukosis, the occurrence of viral diseases leading to mortality was low in the Swedish population of commercial laying hens during the study period. Probably, this was a consequence of vaccination and other prophylactic measures, including screening and eradication of viruses from the breeder population. The differences in occurrence of viral diseases between cage and litter-based housing systems were not as obvious as for parasitic and bacterial diseases. Concerning lymphoid leukosis, the occurrence in the adult laying hens was probably not influenced by the housing system, considering the importance of vertical transmission of the virus, and the development of age resistance [33]. The number of flocks affected by lymphoid leukosis during the study period was unusually high for Sweden, since this disease is not normally present in the Swedish commercial breeding population and commercial laying hens. The outbreaks reported in this study reflect an accidental infection of parents flock of one laying hen hybrid prior to the start of the study.

Cannibalism was one of the main causes of death in laying hens housed in indoor litter-based systems and in free range systems in this study, but it appeared to be a minor problem in caged laying hens. Data from both experimental and commercial flocks from several countries have shown that cannibalism is one of the most severe threats to egg production in both litter-based housing systems and in cages [8-10,16-18,27,34,35]. Several workers have shown that there is a greater risk for a cannibalistic behaviour to occur in larger groups [16,18,36-38]. On Swedish laying hen farms with aviary housing systems, flock sizes between 20,000 and 35,000 birds are not uncommon. Beak trimming, which is a common prophylactic measure against cannibalism in many countries, is not allowed in Sweden according to the Swedish Animal Welfare Act. Consequently, cloacal pecking and cannibalism must be considered to be important health risks in large flocks. The low occurrence of cannibalism in the Swedish cage systems may, at least partly, be explained by the small average group size at the time of study.

Overall, laying hens from flocks housed in litter-based systems and free-range systems were comparatively more frequently submitted for necropsy to SVA during the years 2001–2004, indicating a higher risk for increased mortality in these systems than in cages. Comparisons of mortality, health and performance of laying hens housed in cages and litter-based systems have been performed on an experimental basis (small scale experiments and experimental farms with up to 10 000 birds) by several workers [16,34,39-42] in different countries and results from large-scale investigations comprising commercial laying hens in different housing systems have been reported from Germany [8,10], Switzerland [35,43], Denmark [44] and The Netherlands [45]. The results from the experimental trials show great variations, but the field surveys generally point to increased risk of disease and mortality in litter-based and free-range systems compared to cages agreeing with the findings in this study.

In this study, we have identified several disease problems being associated with the different housing systems. It is, however, important to emphasize that the results reflect the unique situation in Sweden during the years 2001–2004, when the change of housing systems from conventional battery cages was at its peak. Several new types of aviary systems were introduced, and the knowledge and experience of keeping large flocks of laying hens in aviaries was limited. Additionally, during the study period many new egg laying farms were established by people with no or little prior experience of keeping commercial laying hens, and these producers often chose the new aviary systems. Hence, the health status of birds in free range and litter based systems should improve as more experience and knowledge are gained. Preliminary results of necropsies of laying hens after 2004 (not included in this study) indicate that the health situation in the laying hen population has markedly improved [46].

## Conclusion

Data from the present study suggested that during the change in housing systems for laying hens in Sweden in 2001–2004, there were significant differences in bird health between housing systems, with birds in litter-based housing systems and free-range systems showing more health problems than caged laying hens. To increase the safety of the egg production in litter-based housing systems and free-range systems it is necessary to pay much attention to management and preventive measures, such as biosecurity and vaccinations. Adequate education of personnel responsible for running the operations is crucial.

## Competing interests

The authors declare that they have no competing interests.

## Authors' contributions

PEE participated in necropsy work. IV participated in the design of the study and performed the statistical analyses. DSJ and OF participated in designing the study, performed necropsies and drafted the manuscript. All authors read, edited and approved the final manuscript.
